# Regulation of the Stability and Localization of Post-synaptic Membrane Proteins by Liquid-Liquid Phase Separation

**DOI:** 10.3389/fphys.2021.795757

**Published:** 2021-12-16

**Authors:** Tomohisa Hosokawa, Pin-Wu Liu

**Affiliations:** Division of Biological Science, Department of Molecular Biology, Graduate School of Science, Nagoya University, Nagoya, Japan

**Keywords:** liquid-liquid phase separation, neuron, synapse, synaptic plasticity, post-synaptic density

## Abstract

Synaptic plasticity is a cellular mechanism of learning and memory. The synaptic strength can be persistently upregulated or downregulated to update the information sent to the neuronal network and form a memory engram. For its molecular mechanism, the stability of α-amino-3-hydroxyl-5-methyl-4-isoxazolepropionate-type glutamate receptor (AMPAR), a glutamatergic ionotropic receptor, on the postsynaptic membrane has been studied for these two decades. Since AMPAR is not saturated on the postsynaptic membrane during a single event of neurotransmitter release, the number and nanoscale localization of AMPAR is critical for regulating the efficacy of synaptic transmission. The observation of AMPAR on the postsynaptic membrane by super-resolution microscopy revealed that AMPAR forms a nanodomain that is defined as a stable segregated cluster on the postsynaptic membrane to increase the efficacy of synaptic transmission. Postsynaptic density (PSD), an intracellular protein condensate underneath the postsynaptic membrane, regulates AMPAR dynamics *via* the intracellular domain of Stargazin, an auxiliary subunit of AMPAR. Recently, it was reported that PSD is organized by liquid-liquid phase separation (LLPS) to form liquid-like protein condensates. Furthermore, the calcium signal induced by the learning event triggers the persistent formation of sub-compartments of different protein groups inside protein condensates. This explains the formation of nanodomains *via* synaptic activation. The liquid-like properties of LLPS protein condensates are ideal for the molecular mechanism of synaptic plasticity. In this review, we summarize the recent progress in the properties and regulation of synaptic plasticity, postsynaptic receptors, PSD, and LLPS.

## Introduction

Memory formation is a process of the conversion of information from a transient state to a permanent state. Synaptic plasticity, including long-term potentiation (LTP) and long-term depression (LTD), is a cellular mechanism of this process (Malinow and Malenka, 2002; Kauer and Malenka, 2007; Collingridge et al., 2010; Huganir and Nicoll, 2013). Synaptic activity evoked by a learning event triggers transient calcium (Ca^2+^) influx into the postsynaptic site and mediates downstream signals to establish persistent LTP/LTD. The bidirectional regulation and persistence of synaptic plasticity are the essence of memory formation. The interaction between a ligand released from the presynaptic active zone and a receptor on the postsynaptic membrane accomplishes synaptic transmission. Thus, to understand synaptic plasticity, the regulation of the dynamics of postsynaptic membrane proteins is critical as a molecular mechanism. In particular, the dynamics and stability of α-amino-3-hydroxyl-5-methyl-4-isoxazolepropionate-type glutamate receptors (AMPARs) have been studied for more than two decades as major mediators of excitatory synaptic transmission in the mammalian central nervous system (Hayashi et al., 2000; Malinow and Malenka, 2002; Kennedy and Ehlers, 2006; Derkach et al., 2007; Shepherd and Huganirl, 2007; Huganir and Nicoll, 2013). In general, membrane proteins show high mobility in the lipid bilayer membrane with lateral diffusion. However, postsynaptic membrane proteins are anchored by postsynaptic density (PSD), a protein condensate underneath the postsynaptic membrane. PSD is commonly found in excitatory synapses (Palay, 1956; Akert et al., 1969; Harris et al., 1992; Petersen et al., 2003) and stabilized at the postsynaptic site (Shinohara and Hirase, 2009; Tarusawa et al., 2009; Budisantoso et al., 2012; Fukazawa and Shigemoto, 2012; Holderith et al., 2012; Choquet and Triller, 2013). PSD is composed of the intracellular domain of membrane proteins, such as receptors and adhesion molecules, scaffolding proteins, and enzymatic signaling factors (Kennedy et al., 1983; Sugiyama et al., 2005; Sheng and Hoogenraad, 2007). Thus, investigating the interaction between the intracellular domain of AMPAR and PSD proteins and their regulatory mechanisms during synaptic activity is critical for understanding the dynamics of AMPAR and synaptic plasticity.

## The Nanodomain of AMPAR as a Molecular Mechanism of Synaptic Plasticity

To observe the dynamic of AMPAR on the postsynaptic membrane, the single-molecule tracking approach had been performed and revealed that the moving speed of AMPAR was decreased in a neuronal activity-dependent manner (Borgdorff and Choquet, 2002). It has also been reported that the Ca^2+^ influx during LTP and subsequent CaMKII activation reduce the movement of AMPARs and restrict the diffusion area of AMPARs on the postsynaptic membrane (Opazo et al., 2010). This synaptic trapping of AMPAR leads to the formation of several segregated AMPAR clusters on the postsynaptic membrane, which are then defined as the “nanodomains of AMPAR.” AMPAR nanodomains have also been observed with other super-resolution approaches, such as universal point accumulation in the nanoscale topography (uPAINT), stimulated emission depletion (STED) microscopy, and direct stochastic optical reconstruction microscopy (dSTORM) (Nair et al., 2013). Based on these observations, it is known that each synapse contains about 2.5 AMPAR nanodomains with an average length of 77 nm approximately, and around 65% of AMPAR on the entire postsynaptic membrane is concentrated in the nanodomains. Similar structures with unevenly distributed and segregated clusters have also been found in other types of glutamate receptors, such as N-methyl-D-aspartate receptor (NMDAR) (Kellermayer et al., 2018; Goncalves et al., 2020) and metabotropic glutamate receptors (Goncalves et al., 2020), scaffolding proteins, such as PSD-95, guanylate kinase-associated protein (GKAP), Shank3, and Homer1c (MacGillavry et al., 2013), and the adhesion molecule Neuroligin-1 (Haas et al., 2018). This indicates that, in addition to AMPAR, various nanoscale segregated clusters of different protein components exist inside a single PSD structure. Interestingly, a dual-color three-dimensional dSTORM analysis revealed that the localization of the AMPAR nanodomain overlaps with the segregated cluster of PSD-95 at PSD and the presynaptic protein cluster of RIM1/2 (Tang et al., 2016), a marker for presynaptic active zones (Südhof, 2012). Furthermore, the formation of the AMPAR nanodomain by synaptic activity acts as a retrograde signal *via* adhesion molecules to align the localization of presynaptic protein clusters and active zones (Tang et al., 2016). In this manner, synaptic activity mediates the formation of a structure with aligned protein clusters from the PSD to the presynaptic terminal *via* the AMPAR nanodomain and is called a trans-synaptic nanocolumn. Since the ligand binding of AMPAR is not saturated in a single glutamate event (Tong and Jahr, 1994; Liu et al., 1999), this mechanism strengthens the synaptic transmission as “retrograde plasticity” (Tang et al., 2016). Therefore, determining the mechanism of the formation and interaction of the nanodomain/cluster of AMPAR/PSD proteins becomes crucial to understanding synaptic plasticity.

## Liquid-Liquid Phase Separation as a Regulatory Mechanism of PSD

A recent review paper by Groc and Choquet (2020) summarized the movements of glutamate receptors and their mechanism. They discussed the different behaviors between AMPAR and NMDAR during LTP. They also specified the stabilization of AMPAR in PSD *via* the interaction between Stargazin and PSD-95, and the dynamics of NMDAR are regulated by the interaction with CaMKII. However, emerging evidence suggests that the proteins do not just have interaction, but they undergo liquid-liquid phase separation (LLPS) to form protein condensates.

LLPS is a phenomenon that describes the formation of liquid-like condensates as droplets from different types of liquids, such as water and oil. Recent studies have revealed that nucleic acids and/or proteins form condensates inside the cell as compartmentalized droplets in the cytosol (Banani et al., 2017; Boeynaems et al., 2018; Zhang et al., 2020). Unlike an aggregation, the components in the condensates can maintain their dynamics by mixing freely inside the condensate and getting exchanged with the same component molecules from the surrounding environment, such as the cytosol. These condensates have been found to play important roles in neuronal function, such as the development of the central nervous system and the release of neurotransmitters at the presynaptic terminal (Milovanovic et al., 2018; Wu et al., 2020; Hayashi et al., 2021). For PSD proteins, an *in vitro* experiment has first revealed that the representative PSD scaffolding proteins, PSD-95 and SynGAP, undergo LLPS (Zeng et al., 2016). Later, other synaptic proteins, including GKAP, Shank, Homer, and GluN2B, have also been reported to undergo LLPS through their multiplexed interactions and/or interactions through their intrinsically disordered regions (Zeng et al., 2018). These reports raise the possibility that clusters of PSD protein components are formed as LLPS protein condensates.

Postsynaptic density (PSD) has several features that could be explained by the liquid-like properties of LLPS. First, PSD in synapses shows the exchange of component proteins between the inside and outside of PSD (Kuriu et al., 2006; Sharma et al., 2006). PSD also shows the incorporation of cytosolic proteins (Bosch et al., 2014) and the rapid rearrangement of its structure (Blanpied et al., 2008; Kerr et al., 2011; Sun et al., 2021) upon stimulation and Ca^2+^ influx. In addition, although PSD is defined as a highly dense area under electron microscopy, it exhibits multiplexed shapes, such as fenestrated, horseshoe, and segmented (Toni et al., 2001; Borczyk et al., 2019). Similarly, it has been reported that PSD-95 shows perforated or ring-like structures with STED microscopy observation (Broadhead et al., 2016; Masch et al., 2018; Wegner et al., 2018). The LLPS protein condensates have also been found to show the ring-like distribution of their components, especially when they have two different phases inside as core-shell condensate (Gallego et al., 2020; Fare et al., 2021). Nonetheless, the fusing event between two protein condensates is another typical liquid-like property of LLPS. Toni et al. (2001) revealed that the proportion of perforated PSD increased 30 min after LTP induction, and it backs to basal level from 45 to 120 min, which could also be explained by the fusion between two PSDs.

## The PSD LLPS Protein Condensate and the AMPAR Nanodomain

Considering the PSD as an LLPS protein condensate, it comes with the following questions. (1) Can a PSD protein condensate contain several uneven distributions of different component proteins like the clustered nanodomains in PSD? (2) Can the PSD protein condensate be regulated bidirectionally? (3) Can the PSD protein condensate be maintained persistently? The answers have been reported in a recent study. Hosokawa et al. (2021) revealed that Ca^2+^ triggers the persistent formation of nanodomain-like structures in the PSD protein condensate *via* the activation of CaMKII. With purified proteins, the authors found that activated CaMKII by Ca^2+^ influx signal undergoes LLPS and forms a protein condensate with GluN2B, the subunit of NMDAR. This finding is consistent with the observation that CaMKII is incorporated into the PSD from the cytosolic pool during LTP (Bosch et al., 2014). Interestingly, once the CaMKII-GluN2B condensate is formed, it becomes independent of the Ca^2+^ concentration and becomes a permanent protein condensate. This can occur because of the autophosphorylation of CaMKII, which locks CaMKII into an active conformation and maintains its interaction with GluN2B (Bayer et al., 2001; Lisman et al., 2002).

**Figure 1:**
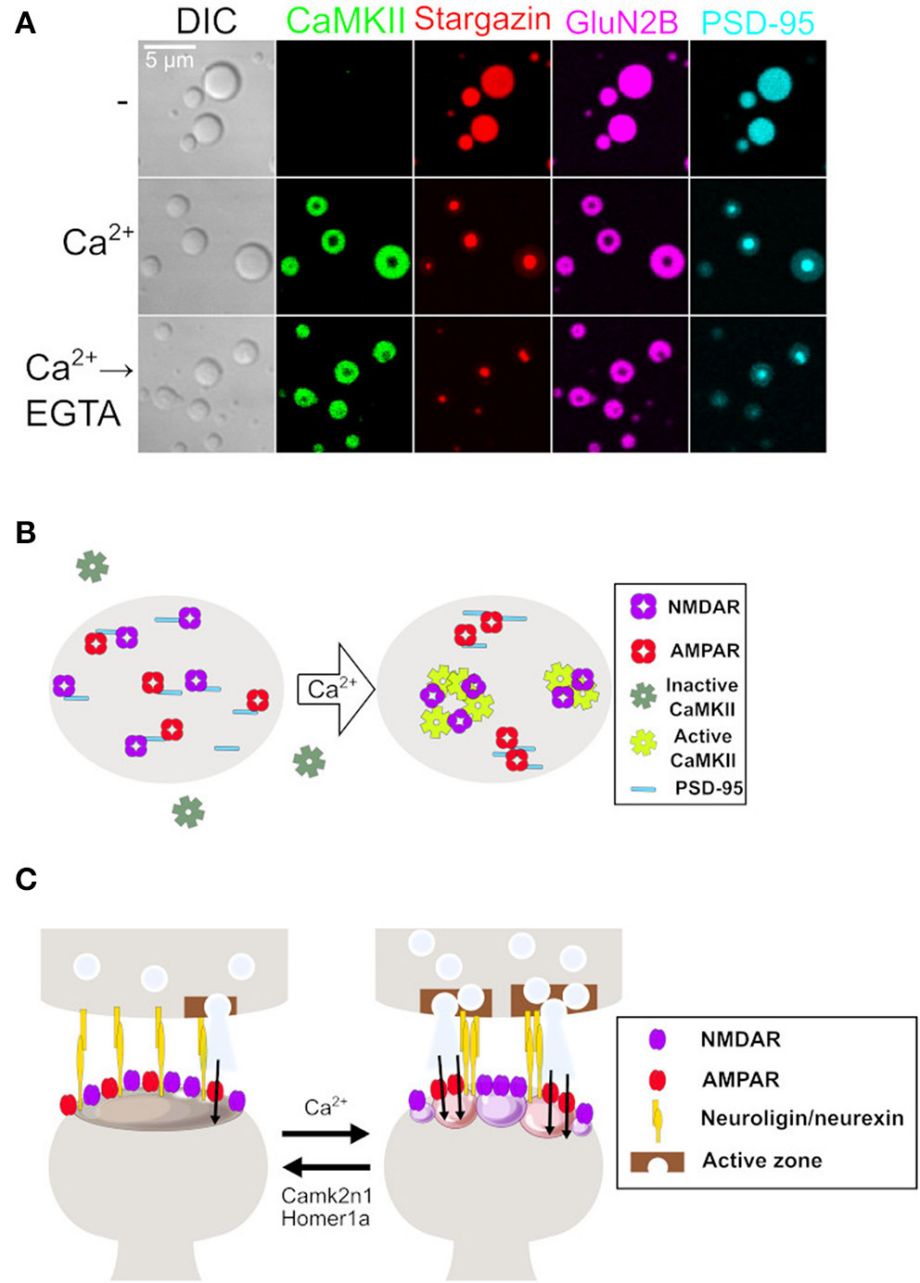
LLPS to synaptic plasticity. **(A)** Microscope images of protein condensates consist of the following four proteins during calcium stimulation. From left to right, confocal images of differential interference contrast and confocal fluorescent images of CaMKII, Stargazin, GluN2B, and PSD-95. Stargazin, GluN2B, and PSD-95 are homogenously distributed before Ca^2+^. Ca^2+^ triggers the incorporation of CaMKII and the formation of a nanodomain-like structure inside of condensate. The condensate is sustained even after the removal of Ca^2+^ by ethylene glycol-bis(β-aminoethyl ether)-N,N,N′,N′-tetraacetic acid (EGTA). Modified from Hosokawa et al. (2021). **(B)** An end view of the postsynaptic membrane. Receptors and PSD proteins in naïve synapses are evenly distributed. Ca^2+^ influx triggers persistent sub-compartmentalization of proteins into the Stargazin-PSD-95 group and CaMKII-GluN2B group, resulting in the formation of the AMPAR nanodomain. Modified from Hosokawa et al. (2021). **(C)** Side sectional view of the synapse. Ca^2+^ influx triggers the formation of AMPAR nanodomains *via* PSD clustering and LLPS. This affects protein assembly in the presynaptic terminal through adhesion proteins as a retrograde signal. In contrast, a dissociation of LLPS by Camk2n1 and Homer1a might act as a mechanism to depress synaptic strength by disrupting the AMPAR nanodomain and PSD clustering. Modified from Hosokawa et al. (2021). LLPS, liquid-liquid phase separation; PSD, postsynaptic density; AMPAR, α-amino-3-hydroxyl-5-methyl-4-isoxazolepropionate-type glutamate receptor.

Since CaMKII is a major component of PSD (Kennedy et al., 1983), the incorporation of CaMKII may be related to the rearrangement of PSD. A previous study has reported that PSD-95 undergoes LLPS and forms an autonomous protein condensate with both GluN2B and Stargazin, an auxiliary subunit of AMPAR (Zeng et al., 2018, 2019), as a possible mechanism for the formation of the basal structure of PSD. Surprisingly, the incorporation of CaMKII into the PSD-95-GluN2B-Stargazin autonomous protein condensate leads to the segregation of the Stargazin-PSD-95 protein condensate from the GluN2B-CaMKII protein condensate, resulting in the formation of a nanodomain-like structure in a single protein condensate (Figure 1A). This explains the formation of the AMPAR nanodomain on the postsynaptic membrane (Figure 1B). Furthermore, Neuroligin-1, a postsynaptic adhesion molecule that clusters with presynaptic neurexin, also segregates together with the Stargazin-PSD-95 protein condensate. It has been known that the cluster formation of Neuroligin-1 induces clustering of presynaptic neurexin, which eventually forms the assembly of an exocytotic apparatus (Dean et al., 2003). Thus, these results suggest a possible mechanism for the formation of AMPAR nanodomains and transsynaptic nanocolumns in a Ca^2+^-dependent manner.

## From LLPS to Synaptic Plasticity

As mentioned above, there are three layers of the mechanisms underlying synaptic plasticity: from bottom to top, the “LLPS” as the regulatory mechanism of PSD, the “PSD” as the regulatory mechanism of the dynamics of AMPAR, and the “dynamics of AMPAR” as the molecular mechanism of synaptic plasticity. However, the molecular mechanisms of synaptic plasticity must satisfy at least two criteria. (1) The mechanism should allow bidirectional regulation since the synapse can be both potentiated and depressed. (2) The mechanism should have a system to maintain its alteration persistently, as synaptic plasticity and memory can be permanent. The three layers of the mechanism satisfy these criteria. Here is a summary of the reported bidirectional regulation (Table 1) and the persistency in each layer.

**Table 1:** Corresponding relationship from LLPS to the synapse.

	**Up-regulation**	**Down-regulation**
LLPS	Condensate with nanodomain-like structure	Dispersion
PSD	Clustering of proteins	Loss of proteins
Receptor	Stabilize as nanodomain	Lateral diffusion
Synapse	Potentiation	Depression

*LLPS-mediated condensation of PSD proteins with nanodomain-like structures corresponds to the clustering of PSD, stabilization of AMPAR as nanodomain, and synaptic potentiation. In contrast, the dispersion of PSD protein condensate corresponds to the loss of scaffolding proteins in the PSD, increased lateral diffusion, and synaptic depression*.

### Regulation and Stability of LLPS Protein Condensate of PSD Proteins

For the bidirectional regulation of LLPS protein condensate, the Ca^2+^ signal triggers the persistent formation of the CaMKII-GluN2B protein condensate (Hosokawa et al., 2021). In contrast, the endogenous proteins, Camk2n1 and Homer1a, act as dissociation factors against PSD protein condensates in different ways (Zeng et al., 2018; Hosokawa et al., 2021). While Camk2n1 disrupts the Ca^2+^-dependent CaMKII-GluN2B protein condensate, Homer1a disrupts the Ca^2+^-independent protein condensate, which is composed of GluN2B, SynGAP, PSD-95, GKAP, Shank3, and Homer3. Homer1a has been reported to be related to synaptic depression (Diering et al., 2017). Nonetheless, LLPS protein condensate is known to be a stable membrane-less protein condensate that enables the formation of a stable structure for more than several days (Ray et al., 2020). Being an LLPS protein condensate allows PSD to overcome the limitation of metabolic turnover of PSD proteins (Cohen et al., 2013) by exchanging the component proteins between inside and outside of the PSD. Also, LLPS protein condensates are possibly acting as a filter of molecules (Alberti et al., 2019) to protect their components from other proteins, such as proteases. Taken together, LLPS protein condensate has ideal properties to guarantee bidirectional regulation and persistence.

### Regulation and Stability of PSD

Postsynaptic density (PSD) modifies the dynamics of AMPAR through enlargement and shrinkage. It is known that the size of PSD is correlated with the number of AMPAR nanodomains, the size of dendritic spines, and synaptic strength (Harris et al., 1992; Noguchi et al., 2005; Nair et al., 2013; Meyer et al., 2014). The Ca^2+^ influx during LTP mediates an increase in the size and complexity of PSD (Sun et al., 2021), and the growth of PSD (Harris, 2020). On the contrary, LTD induction results in a loss of PSD components, such as PSD-95 (Bingol and Shang, 2011; Compans et al., 2021). To maintain the persistency, PSD components also show protein exchange between the inside and outside of PSD, which contributes to its homeostasis. Also, the dendritic spine as a proxy for PSD survives for more than 1 month in general (Grutzendler et al., 2002). Taken together, PSD is a stable structure but regulated bidirectionally in response to synaptic activity.

### Dynamics of AMPAR and Synaptic Plasticity

The AMPAR nanodomain is known to be formed by synaptic activity (Opazo et al., 2010; Nair et al., 2013). In addition, LTD induction results in an increase in AMPAR lateral diffusion, which may result in the disruption of the nanodomain (Chowdhury and Hell, 2019; Compans et al., 2021). It has been reported that more than 60% of AMPAR nanodomains persist for at least 45 min (Nair et al., 2013). Even though a prolonged observation is lacking due to technical difficulties, considering that nanodomains are commonly observed in unstimulated neurons, AMPAR nanodomains must be able to be long-lasting structures. Taken together, LLPS as a bidirectionally regulated persistent protein condensate explains synaptic plasticity by regulating PSD and the dynamics of AMPAR on the postsynaptic membrane (Figure 1C).

## Discussion

In this review, we focused on the regulation of AMPAR on the postsynaptic membrane by the intracellular PSD LLPS protein condensate. LLPS protein condensate determines the stability of AMPARs on the postsynaptic membrane by mediating persistent condensation and dispersion of PSD proteins. The transient information, such as synaptic activity-mediated Ca^2+^ influx, is converted into a persistent structure as the protein condensate and the AMPAR nanodomain. This can be accomplished only by the protein condensate, not by a single protein. This is because the protein itself cannot persistently maintain information due to diffusion, de-modification, and degradation. Therefore, the LLPS protein condensate, including PSD clusters and AMPAR nanodomains, might be the minimum unit of memory as “molecular memory engram,” which means the molecular evidence of memory engram (Liu et al., 2012).

## Author Contributions

TH contributed to writing and revising the manuscript. P-WL participated in revising and making illustrations. All authors contributed to the article and approved the submitted version.

## Funding

The Takeda Science Foundation and Grants-in-Aid for Scientific Research JP17K14947, JP18KK0421, and JP19K06885 from MEXT Japan and CREST JPMJCR20E4 from the Japan Science and Technology Agency to TH. Grants-in-Aid for Research Activity Start-up 20K22685 and Grants-in-Aid for Early-Career Scientists 21K15188 from the Japan Science and Technology Agency to P-WL.

## Conflict of Interest

The authors declare that the research was conducted in the absence of any commercial or financial relationships that could be construed as a potential conflict of interest.

## Publisher's Note

All claims expressed in this article are solely those of the authors and do not necessarily represent those of their affiliated organizations, or those of the publisher, the editors and the reviewers. Any product that may be evaluated in this article, or claim that may be made by its manufacturer, is not guaranteed or endorsed by the publisher.

## Abbreviations

AMPA: α-amino-3-hydroxy-5-methyl-4-isoxazolepropionic acid
AMPAR: AMPA-type glutamate receptor
CaMKII: Calcium/Calmodulin-dependent kinase II
dSTORM: direct stochastic optical reconstruction microscopy
LLPS: Liquid-Liquid Phase Separation
LTP: Long-term potentiation
LTD: long-term depression
NMDA: N-methyl-D-aspartate
NMDAR: NMDA-type glutamate receptor
PSD: Postsynaptic Density
STED: stimulated emission depletion microscopy
uPAINT: universal point accumulation in nanoscale topography.
